# Successful Management of Two Consecutive Pregnancies With Maternal–Fetal Phenylketonuria: Lessons From Clinical Practice

**DOI:** 10.1002/jmd2.70054

**Published:** 2025-12-03

**Authors:** Per Lundkvist, Karin Blom Malmberg, Linda Lindström, Andreas Kindmark

**Affiliations:** ^1^ Department of Medical Sciences Uppsala University Uppsala Sweden; ^2^ Geriatrics, Rehabilitation Medicine and Pain Centre Uppsala University Uppsala Sweden; ^3^ Department of Women's and Children's Health Uppsala University Uppsala Sweden

**Keywords:** dietary management, maternal phenylketonuria, maternal–fetal PKU, medical nutrition therapy, metabolic control, phenylalanine, phenylketonuria, pregnancy

## Abstract

Phenylketonuria (PKU) is an autosomal recessive disorder caused by a deficiency of phenylalanine hydroxylase (PAH), leading to the accumulation of phenylalanine (Phe) and an increased risk of developmental disorders. Treatment involves a Phe‐restricted diet, amino acid supplements, and for a subset of patients, a tetrahydrobiopterin (BH4) chaperone. Managing PKU during pregnancy is challenging due to changing protein and energy needs, stricter Phe control, nausea, and unpalatable supplements. In rare cases of simultaneous maternal and fetal PKU, Phe tolerance may increase less throughout gestation, raising the demands on the patient and caregivers. There are few reports and no guidelines on the management of PKU during pregnancies in which both mother and fetus have PKU, hereafter referred to as maternal‐fetal PKU (mfPKU). This report outlines our approach for successfully managing two consecutive mfPKU pregnancies. We emphasize a patient‐centered approach, focusing on patient education and close collaboration with a multidisciplinary metabolic team. This involves regular monitoring of body weight, blood Phe levels, and calorie intake through an online food diary to tailor individual recommendations for natural protein restriction and amino acid supplements.

## Summary

- Maternal–fetal phenylketonuria (mfPKU) is a rare clinical situation with very limited guidance in the literature, underscoring the need for individualized metabolic strategies.
- Two consecutive mfPKU pregnancies were managed successfully through preconception stabilization, lowered phenylalanine (Phe) targets, and frequent dried blood spot measurements.
- Both pregnancies showed substantial increases in Phe tolerance despite absent fetal PAH activity, indicating that the regulation of Phe tolerance during gestation extends beyond fetal enzymatic capacity.
- Continuous collaboration within a multidisciplinary metabolic team, together with detailed dietary monitoring and active patient participation, enabled sustained metabolic control.
- These observations illustrate that rigorous, individualized metabolic management can support favorable pregnancy outcomes even in the rare setting of mfPKU.

## 1 Introduction

Phenylketonuria (OMIM#261600) is an autosomal recessive metabolic disorder characterized by a deficiency in the enzyme PAH, leading to the accumulation of Phe and subsequent neurological complications [1]. Affecting 1:10000 live births in Europe, PKU is the most prevalent inborn error of amino acid metabolism [2]. Untreated PKU may lead to severe developmental abnormalities. Strict dietary management and BH4 chaperone treatment in BH4‐responders allow for near‐normal growth and development [1, 3, 4]. The mother's residual PAH activity determines her individual pregestational Phe tolerance. In pregnancy, Phe tolerance generally increases as tissues grow, and PAH activity develops in the liver of the fetus.

## 2 Maternal Considerations in Phenylketonuria

Pregnancy in women with PKU poses a challenge to prospective mothers and caregivers alike [5]. Achieving and maintaining Phe within the recommended target range during pregnancy (120–360 μmol/L in current guidelines) [6, 7] requires strict metabolic governance, generally warranting months preconception of dietary training [8]. The medical nutritional therapy (MNT) invariably consists of a Phe‐restricted diet, Phe‐free L‐amino acid supplements, and, in BH4‐responders, a BH4 chaperone. The individuals' pregestational Phe tolerance varies greatly with PKU phenotype [9], setting the baseline for natural protein allowance during pregnancy planning (or at conception if the pregnancy is not planned). The expected increase in Phe tolerance during gestation can also vary with the PKU status of the fetus [10], multiparity [11] and the mother's bodyweight [12].

Protein requirements increase progressively throughout gestation [13]. Active transport of amino acids across the placenta, together with placental synthesis and turnover of amino acids, ensures that the fetus receives sufficient substrates and energy for normal growth and development [14]. In maternal PKU (mPKU), maintaining the balance between toxic Phe levels and nutrient scarcity in the developing fetus is particularly challenging. Also, catabolic periods, such as those induced by illness or hyperemesis, can further elevate Phe levels in mPKU [15]. Phe levels often peak during the latter part of the first trimester [4, 16], when hyperemesis typically culminates. Also, Phe concentrates significantly in fetal tissues via placental transfer [17], exerting incrementally teratogenic effects in mPKU [18], known as maternal PKU syndrome (mPKU‐S). Adverse outcomes associated with mPKU‐S can be severe and include fetal growth restriction, preterm birth, low birth weight, microcephaly, impeded neurodevelopment, and congenital heart disease [7, 19]. The association between Phe levels > 600 μmol/L persisting beyond 10 weeks of gestation and impaired fetal neurodevelopment is well established [6]. Although the pathomechanisms of high Phe remain incompletely elucidated, likely culprits include deficiencies of displaced substrates and neuronal dysfunction from Phe toxicity [20].

Nutritional needs, including natural protein requirements (or Phe tolerance), increase as mother and fetus grow. By gestational week 13, a non‐affected fetus has significant PAH enzyme activity [21] and can metabolize Phe, while a fetus deficient in PAH cannot [6]. Hence, maternal Phe tolerance in mPKU is expected to increase during the second and third trimester both from overall anabolism and fetal PAH enzyme activity. However, a smaller increase in Phe tolerance than expected can indicate PKU in the fetus [10].

Consequently, to prevent PKU‐associated fetopathy in mfPKU, extra metabolic diligence seems prudent. How to achieve this is not defined in existing guidelines, and few case reports exist. Considering this clear unmet need for metabolic team guidance, we share our experience of caring for two consecutive mfPKU pregnancies.

## 3 Maternal PKU Monitoring

At Uppsala University Hospital, a metabolic team consisting of a dietician, physician, and nurse is responsible for the care of PKU patients. All female PKU patients are informed of the implications of pregnancy in PKU as teens, and the information is then repeated regularly. During pregnancy planning, Phe is monitored by dried blood spot (DBS) analyses three times per week, based on which the dietician recommends natural protein ingestion and amino acid substitution adjusted to body weight.

## 4 Clinical Case Synopsis: Maternal‐Fetal Phenylketonuria

A Swedish woman, born in 1992, was diagnosed with PKU through neonatal screening, with a clinical classic phenotype and mutation analysis results with a c.674C>G, p.(Pro225Arg) and c.1222C>T, p(Arg408Trp) genotype of the PAH gene. The Arg408Trp mutation has an allelic phenotype value (APV) of 0.5 in the BioPKU database [22], while the Pro225Arg mutation has been described previously in PKU patients [23] and is labeled in the Qiagen Franklin [24] as a non‐synonymous variant in a functional domain, where the Pro225Leu at the same amino acid position is a well‐known pathogenic variant associated with classical PKU. Monitored at Uppsala University Hospital's pediatric metabolic unit until adulthood, she was transferred to the adult metabolic unit at age 20. In the following years, the Phe level ranged between 400 and 700 μmol/L, until age 25, when she expressed an intention to conceive. Notably, the intended father also had PKU with mutation analysis results of c.1222C>T, p(Arg408Trp) and c.1315 + 1G>A, p.,(?) (BioPKU predicted Classic PKU, with genotypic phenotype value (GPV) of 0.5), predicting any offspring PAH‐deficient as well. Thus, the maternal Phe tolerance would be expected to be decreased more than in a pregnancy with a mother with PKU where the fetus did not have a PAH deficiency [10].

At this time, the recommended daily total protein intake was estimated to be 81–89 g per day, calculated based on a daily requirement of 1.1–1.2 g/kg and a pregestational body weight of 74 kg (adjusted from overweight to BMI 25 kg/m^2^). The recommended energy intake was approximately 2200 kcal/day in the first trimester [25] and increased gradually based on her weight gain.

The preconception Phe target was set to 120–360 μmol/L, and MNT intensified to 8 g of natural protein and 70 g of amino acid supplements. It was difficult for her to tolerate a larger amount of Phe‐free L‐amino acid supplements at that time. Once Phe levels had been stable within the target range for 3 months, contraceptive methods ceased, and attempts at pregnancy ensued. However, a year of unsuccessful efforts led to a diagnosis of primary infertility (anti‐Müllerian hormone level of 31 pg/mL), and subsequent assisted reproduction. Controlled ovarian stimulation cycles with letrozole, an aromatase inhibitor, resulted in successful conception on the sixth attempt.

**FIGURE 1:**
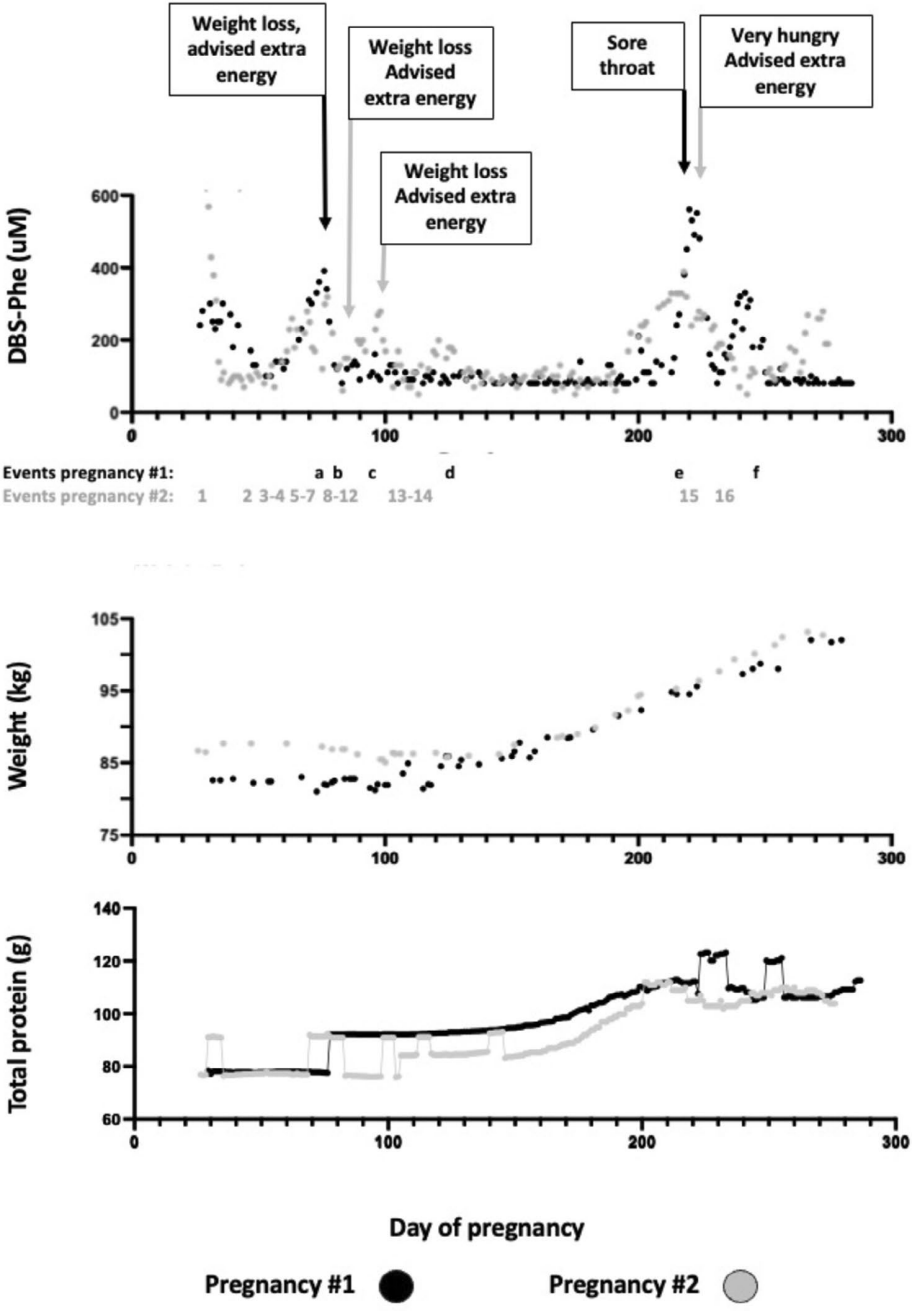
Measurements during the pregnancies. Changes in DBS‐Phe, maternal body weight and total protein intake summarizing total protein intake over the course of the two consecutive mfPKU pregnancies.

After confirming a viable pregnancy, the Phe target range was discussed with the Swedish national metabolic medicine experts at Karolinska University Hospital. Based on a lower expected Phe tolerance due to impaired fetal PAH activation, the target range was lowered to 120–200 μmol/L to avoid an accumulation of toxic Phe levels in fetal circulation. This change was implemented from day 33 of the pregnancy. An intensified ultrasonography protocol was put in place for the early detection of congenital heart defects and the monitoring of fetal development. DBS Phe monitoring was conducted 3–5 times per week. At the time, Phe levels were not quantified below 80 μmol/L, but instead only reported as “Phe < 80”, making treatment decisions based on low Phe values challenging. The turnaround time for DBS Phe results varied between one and six days (mean 2, 4 days, SD 1, 3 days). The goal was to empower the patient to actively participate in her own treatment in close collaboration with the medical team. An online nutritional calculation program, Dietist Net, allowed the patient to calculate intake of nutrients, energy and dietary protein, but unfortunately not specifically phenylalanine, and share a food diary with the dietician several times a week. The dietician then gave tailored intake recommendations based on current energy and protein balance, body weight, and DBS Phe levels. These specified the recommended total intake of natural proteins and amino acid supplements. A graph of the patient's total protein intake is presented in Figure 1, and a detailed breakdown of natural versus synthetic protein sources is shown in Supplementary Figure S1. Preventing catabolism was important, and the threshold to start the antiemetic Meclizine or extra nutritional support was low. A healthy daughter was delivered at pregnancy week 41 (3685 g, 51 cm).

A wish for a second pregnancy was expressed a year later but was advised against at that time, due to the then ongoing Covid pandemic, where after an unplanned pregnancy was detected half a year later. Phe values rapidly declined to the recommended range within 1 week after test‐confirmed pregnancy. A healthy daughter was delivered at term (3670 g, 52 cm).

DBS Phe reached an early peak at pregnancy week 10 in both pregnancies (see Table *1* for events influencing protein metabolism). Phe later peaked again during the third trimester, coinciding with a fever spell and vomiting respectively during pregnancy 1, and increased hunger as well as Covid vaccination during pregnancy 2.

**TABLE 1:** Events during pregnancies.

Day of pregnancy #1	Event pregnancy #1		Day of pregnancy #2	Event pregnancy #2
		1	Day 29–35	Extra PKU Easy liquid added due to weight loss and decreased registered energy intake
		2	Day 47	Slight nausea, but no vomiting
		3	Day 54	Recommended to exchange 1 PKU Easy liquid to 20 tablets Phlexy 10 in the evening if nauseous
		4	Day 55	Slight nausea, but no vomiting
		5	Day 62	Received second dose of Covid vaccine
		6	Day 63	Reaction after vaccination with fever, headache, muscle aches, recommended paracetamol
		7	Day 64	Feeling OK again—protein recommendations adjusted due to late incoming Phe results
		8	Day 70–76	Added one PKU Easy Liquid 15
Day 74	Weight loss. No nausea. Recommended extra energy (Nutrical)			
		9	Day 75	Slight nausea, but no vomiting
		10	Day 77–83	Added Phlexy 10, 20 tablets
		11	Day 78–89	Added extra energy
		12	Day 79	Weight loss
Day 81	Target Phe changed to 80–150 μmol/L			
Day 94–97	Cold, weight loss, vomiting			
		13	Day 98–103	Weight loss. Added Phlexy 10, 20 tablets
		14	Day 105–113	No weight gain. Added Phlexy 10, 20 tablets
Day 126	Sore throat and fever. Recommended Calogen and Nutrical if low food intake			
Day 216–223	Sore throat and fever. Recommended Calogen and Nutrical if low food intake			
		15	Day 217–223	Very hungry. Wakes up at night to eat. Recommended extra energy intake
		16	Day 229	Received third dose of Covid vaccine
Day 250	Vomiting			

*Note:* Events during the pregnancies that can impact protein metabolism.

Maternal body weight differed by 8.4 kg at 26 versus 29 days of gestation (pregnancy 1 and 2: 78.3 kg vs. 86.7 kg) and later converged to follow a similar trajectory from pregnancy week 17 onwards.

Dietary protein allowance and Phe tolerance were comparable across the two pregnancies. At gestational week 8, intake levels were 8 g protein (400 mg Phe, calculated as 50 mg Phe/1 g of natural protein) and 6.5 g protein (325 mg Phe), respectively. Requirements began to increase around week 20 and reached their maximum at week 28, with peak values of 27.7 g protein (1385 mg Phe) and 28 g protein (1400 mg Phe). Thereafter, protein allowance and Phe tolerance declined progressively until delivery: 24 g (1200 mg Phe) 19 g (950 mg Phe), respectively. Overall, this represented a maximum increase of approximately 346% and 431% in the respective pregnancies.

The average daily requirement of total protein intake was lower during the first 28 weeks of the second pregnancy compared with the first. This difference is accounted for by a lower allowance of Phe‐free amino acid supplements during pregnancy 2.

Both children were diagnosed with PKU, and the PAH genotypes of the children were Pro225Arg/Arg408Trp (same genotype as the PKU mother with a classical PKU phenotype) and Arg408Trp/Arg408Trp (BioPKU GPV 0,5, Classical PKU), respectively. Both children generally followed their expected growth trajectories for height, weight and head circumference (Supplementary Figure S2), and achieved their developmental milestones during routine follow‐up.

These pregnancies demonstrate the effectiveness, and challenges, of meticulous maternal metabolic control and surveillance in mitigating the inherent genetic risks that mfPKU poses to fetal health.

## 5 Discussion

Hyperphenylalaninemia (HPA) is the hallmark of PKU, necessitating early MNT initiation to preclude neurocognitive impairment of the fetus during pregnancy in women with PKU. While challenging, controlling Phe levels during the vulnerable early phases of fetal development is essential to avoid congenital birth defects. The PAH activity and growth requirements of the mother and fetus are the main determinants of Phe tolerance during pregnancy. Hence, women with little or no PAH activity (classical PKU) generally tolerate a lower daily intake of natural protein than those with substantial residual enzymatic capacity (as in mild HPA). In the rare cases of simultaneous maternal and fetal PKU, tolerance is reported to be even lower [10, 26].

We present a case of a mother with classic PKU and two consecutive, successful pregnancies with the fetus having PKU as well. Once the subject made the pregnancy plans known, strict MNT was employed, with a stable Phe target level between 120 and 360 μmol/L for 3 months prior to conception. This range was subsequently lowered to 120 and 200 μmol/L.

Managing a restrictive diet and frequent Phe testing can be demanding for women with PKU who are planning pregnancy. In this case, stabilizing Phe between 120 and 360 before conception and 120–200 during gestation generally worked well. However, we believe that the timing of preconceptual dietary adjustments should be tailored to each individual's ability to tolerate a less appetizing diet.

The trajectories of natural protein/Phe tolerance were remarkably similar in both pregnancies. Subsequent to positive pregnancy tests, Phe was measured frequently in line with the current iteration of European PKU guidelines [6], and Phe was generally well controlled throughout both gestational periods. The association between a high measuring frequency and low Phe concentration is previously reported on by Yildiz and Sivri [16]. In our case, Phe reached an early peak at pregnancy week 10 of both pregnancies, when pregnancy‐associated nausea is most common, as previously observed in mPKU by Nielsen et al. [4].

Maternal body weight was 8.4 kg higher at the onset of the second pregnancy, a factor previously reported to be associated with low Phe tolerance increase during gestation [12]. In contrast, our data show comparable increases in Phe tolerance across both pregnancies. This apparent discrepancy may be explained by a lower mean intake of Phe‐free amino acid supplements during the first 28 weeks of pregnancy 2.

The second pregnancy was unplanned, with unsuitably high Phe levels when it was detected. Once the metabolic team was informed, this prompted an immediate shift in dietary regime and commencement of frequent DBS Phe measurements (in pregnancy week 5), rapidly decreasing Phe values to within target range (120–200). Previous studies indicate that Phe levels brought to target range prior to pregnancy week 10 are sufficient for near normal fetal development in mPKU [6], based on our results this may not differ in the rare cases of mfPKU.

During both pregnancies, Phe tolerance increased substantially (985 mg, 346% and 1075 mg, 431%). Total daily natural protein allowance/Phe tolerance is previously reported to increase less in cases of mfPKU [10], attributed to low or non‐existent fetal PAH activity (see Table *2* for comparison with previous reports). In contrast, the increase in Phe tolerance in our two cases resembles mPKU more than mfPKU in the paper by Kohlschütter et al. [10]. Assuming that combined fetal and maternal PAH activity and growth demands determine Phe tolerance, these findings appear paradoxical. However, Hozyasz et al. have reported a smaller increase in Phe tolerance in twin compared with singleton PKU pregnancies [11]. This indicates that the regulation of Phe tolerance during pregnancy is more complex than can be explained by fetal and maternal growth demands and enzymatic activity alone.

**TABLE 2:** Comparison of Phe tolerance increase during pregnancy in mPKU and mfPKU.

Author	Case description	Maternal phenotype	Maternal genotype^a^	Fetal PKU/phenotype (genotype)^a^	Daily Phe tolerance increase (mg, %)	Measuring frequency	Target range
Kohlschütter et al. 2009	3 women 3 pregnancies 2 mPKU 1 mfPKU	3 cPKU	p.I94del/p.P281L p.I65T/p.R408W p.R408W/p.R261Q	cPKU (p.R408W/p.R408W)	mPKU 1300 mg, 325% mfPKU 0‐200 mg, 0%–50%	NR	100–250
Yildiz, Sivri 2019	32 women 71 pregnancies 45 live births 33 mPKU 12 mfPKU	18 cPKU 6 mild–moderate PKU 5 HPA 3 mild HPA	21 variants in 54 alleles. Most common variants were c.782G>A and c.1066‐11G>A.	3 cPKU 1 mild–moderate PKU 3 HPA 5 mild HPA	NR	0.71/week on average	NR
Ugalde‐Abiega et al. 2023	1 woman 1 pregnancy	cPKU	L348V/IVS1nt5	Moderate–severe PKU	NR (no dietary change)	Every 15 d	120–360
Hozyasz et al. 2020	3 women each with 1 singleton 1 twin pregnancy	3 cPKU	Q383X/R408W EX3DEL/EX3DEL R281L/R408W	No	Singleton/twin Patient 1: 579%/468% Patient 2: 674%/261% Patient 3: 427%/236%		
Caletti et al. 2020	7 women 10 pregnancies	5 cPKU 1 mild HPA 1 mild PKU/mild HPA	R261Q/DEL E5 IVS8‐7A>G/I65TIVS4 + 5G>T/R158QDEL E3/R158Q P281L/A165P R261Q/A403V E178G/R261Q	No	cPKU 297 mg, 107% mild HPA 597 mg, 17%	1–2/week	120–250
Zolkowska Hozyasz 2019	1 woman 2 pregnancies	cPKU	p.R408W/p.R408W	No	551% 650%	2–3/week	120–360
Our Case	1 woman 2 pregnancies 2 mfPKU	cPKU	c.674C>G, p.(Pro225Arg)/c.1222C>T, p.(Arg408Trp)	2 cPKU Pro225Arg/Arg408Trp Arg408Trp/Arg408Trp^b^	985 mg, 346% 1075 mg, 431%	3–5/week	120–360 120–200^c^

Abbreviation: NR = Not reported.

^a^ Variant nomenclature is reported as in the original references to avoid incorrect reinterpretation. HGVS formatting was applied only where it was used in the source material.

^b^ BioPKU GPV 0,5, predicted classical PKU “Approximation from Phe 50 mg per 1 g natural protein”.

^c^ Implemented from day 33 of pregnancy 1.

In this case, strict metabolic management led to successful term deliveries of two healthy infants without complications related to maternal PKU. Our approach relied on individualized targets, frequent Phe monitoring, and close patient engagement within a multidisciplinary metabolic team. These results suggest that, even in the rare scenario of maternal–fetal PKU, rigorous metabolic control can effectively mitigate genetic and metabolic risks. At the time of writing, both children continue to demonstrate normal growth and development at 4 years 6 months and 2 years 8 months of age, respectively.

## 6 Conclusions

These results illustrate that individualized, multidisciplinary metabolic management in mitigating the genetic risks of maternal–fetal PKU and emphasize the value of specialized maternal–fetal PKU programs to optimize reproductive outcomes in affected couples.

## Funding

The authors have nothing to report.

## Consent

All procedures followed were in accordance with the ethical standards of the responsible committee on human experimentation (institutional and national) and with the Helsinki Declaration of 1975, as revised in 2000. Informed consent was obtained from all patients for being included in the study. Details of the contributions of individual authors.

Per Lundkvist wrote the first draft of the manuscript and was responsible for the majority of writing. Karin Blom Malmberg contributed to sections on diet and Linda Lindström to sections on pregnancy. Andreas Kindmark conceived of the case report, collected the data, ensured compliance with ethical guidelines, obtained necessary approvals, revised the manuscript, and generated figures and visual representations of the data. All authors contributed to writing and reviewing the final manuscript.

## Conflicts of Interest

The authors declare no conflicts of interest.

## Supporting information


**Figure S1:** Protein sources, body weight and DBS‐Phe during the pregnancies changes in DBS‐Phe, maternal body weight and total protein intake summarizing intake of total protein, broken down into natural proteins and amino acid supplements over the course of the two consecutive mfPKU pregnancies.

**Figure S2:** Child growth trajectories of head circumference, height and body weight for 24 months.

## Data Availability

The data that support the findings of this study are available from the corresponding author upon reasonable request.
