# Supplementary figures and images for: Successful Management of Two Consecutive Pregnancies With Maternal–Fetal Phenylketonuria: Lessons From Clinical Practice
Source: JIMD Rep. 2025 Dec 3;67(1):e70054. doi: 10.1002/jmd2.70054 (PMC12674843; doi:10.1002/jmd2.70054)

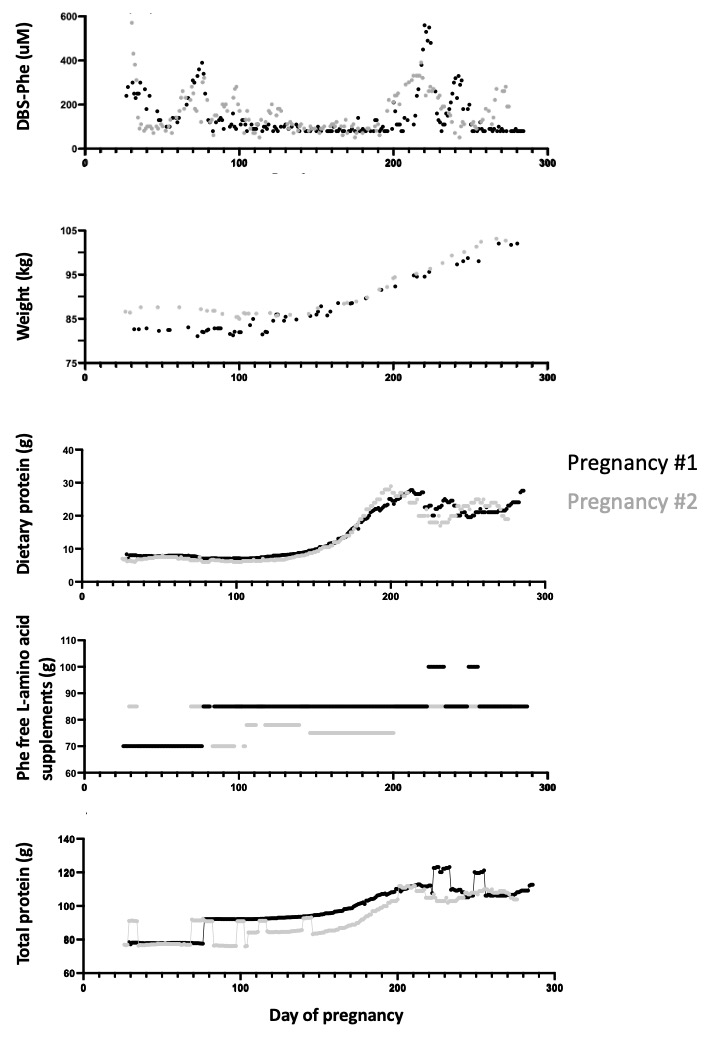

Supplement: Supplementary file 1 — Figure S1: Protein sources, body weight and DBS‐Phe during the pregnancies changes in DBS‐Phe, maternal body weight and total protein intake summarizing intake of total protein, broken down into natural proteins and amino acid supplements over the course of the two consecutive mfPKU pregnancies. [file JMD2-67-e70054-s002.jpeg]
