# Supplementary material for: Successful Management of Two Consecutive Pregnancies With Maternal–Fetal Phenylketonuria: Lessons From Clinical Practice
Source: JIMD Rep. 2025 Dec 3;67(1):e70054. doi: 10.1002/jmd2.70054 (PMC12674843; doi:10.1002/jmd2.70054)
Supplement: Supplementary file 2 — Figure S2: Child growth trajectories of head circumference, height and body weight for 24 months. [file JMD2-67-e70054-s001.pdf]

Personnummer	Efternamn	Förnamn Pregnancy #1	Girl ♀

Moder

Vikt kg	Längd cm	Huvudomf cm

Fader

Vikt kg	Längd cm	Huvudomf cm

Födelseuppgifter

Graviditetstid, veckor + dagar		
Ålderskorrektion, veckor		
Vikt kg	Längd cm	Huvudomf cm

Prematurkurvor

För barn födda före graviditetsvecka 37 använd nedanstående kurvor tills barnet är motsvarande 40 veckor. Använd därefter de ordinarie kurvorna med ålderskorrektion för prematuritet.

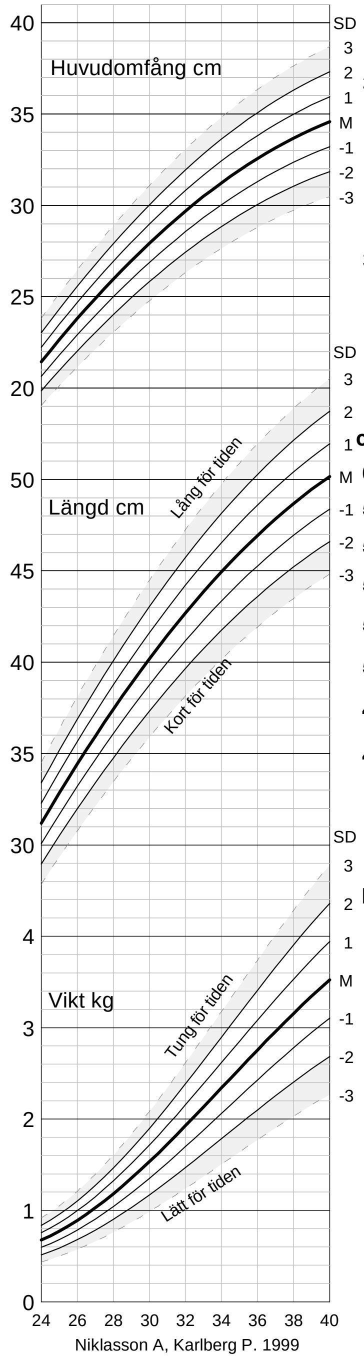

Niklasson A, Karlberg P. 1999

Tillväxtkurvor

För vikt, längd och huvudomfång är medelvärden jämte standardavvikelser (±1 SD, ±2 SD, ±3 SD) angivna för varje ålder. För en normalpopulation ligger tvärsnittsmässigt 67% inom ± 1 SD och 95% inom ± 2 SD. Med värden från upprepade tillfällen införda kan man se om barnets tillväxt följt de kanaler som bildas mellan SD-linjerna.

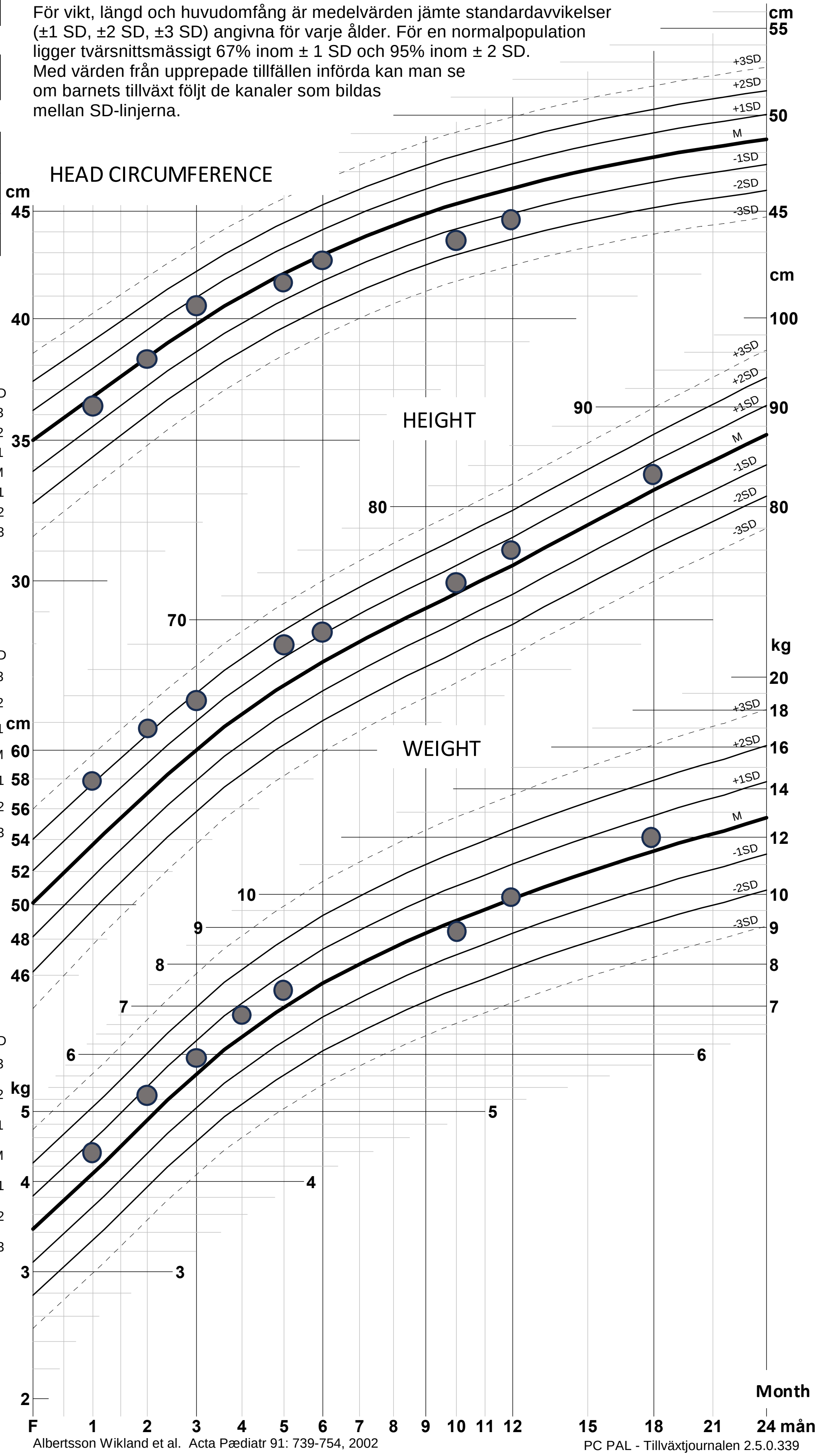

Albertsson Wikland et al. Acta Pædiatr 91: 739-754, 2002

PC PAL User, PC PAL Company - 16-01-2014 13:41:19

PC PAL - Tillväxtjournalen 2.5.0.339

Personnummer	Efternamn	Förnamn Pregnancy #2	Girl ♀

Moder

Vikt kg	Längd cm	Huvudomf cm

Fader

Vikt kg	Längd cm	Huvudomf cm

Födelseuppgifter

Graviditetstid, veckor + dagar		
Ålderskorrektion, veckor		
Vikt kg	Längd cm	Huvudomf cm

Prematurkurvor

För barn födda före graviditetsvecka 37 använd nedanstående kurvor tills barnet är motsvarande 40 veckor. Använd därefter de ordinarie kurvorna med ålderskorrektion för prematuritet.

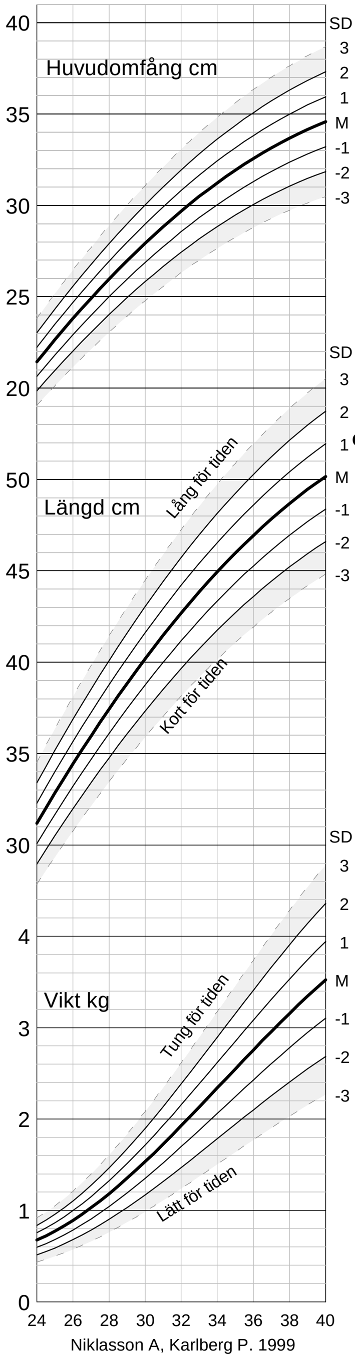

Niklasson A, Karlberg P. 1999

PC PAL User, PC PAL Company - 16-01-2014 13:41:19

Tillväxtkurvor

För vikt, längd och huvudomfång är medelvärden jämte standardavvikelser (±1 SD, ±2 SD, ±3 SD) angivna för varje ålder. För en normalpopulation ligger tvärsnittsmässigt 67% inom ± 1 SD och 95% inom ± 2 SD. Med värden från upprepade tillfällen införda kan man se om barnets tillväxt följt de kanaler som bildas mellan SD-linjerna.

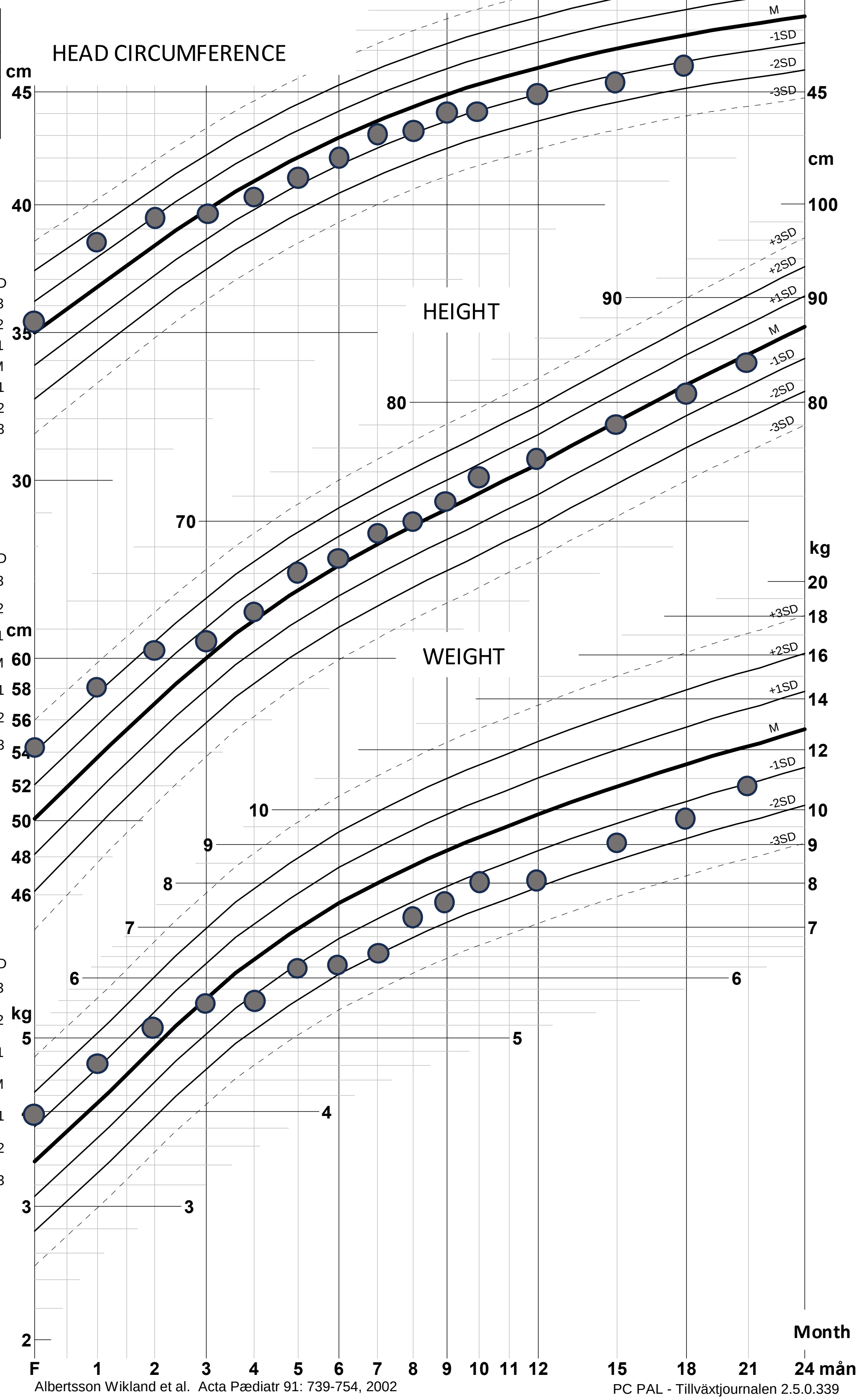

Albertsson Wikland et al. Acta Pædiatr 91: 739-754, 2002
